# Synthesis, crystal structures, HF-EPR, and magnetic properties of six-coordinate transition metal (Co, Ni, and Cu) compounds with a 4-amino-1,2,4-triazole Schiff-base ligand†

**DOI:** 10.1039/c9ra10851c

**Published:** 2020-03-31

**Authors:** Ya-Jie Zhang, Lei Yin, Jing Li, Zhao-Bo Hu, Zhong-Wen Ouyang, You Song, Zhenxing Wang

**Affiliations:** Wuhan National High Magnetic Field Center & School of Physics, Huazhong University of Science and Technology Wuhan Hubei 430074 P. R. China zxwang@hust.edu.cn; State Key Laboratory of Coordination Chemistry, School of Chemistry and Chemical Engineering, Nanjing University Nanjing 210023 P. R. China yousong@nju.edu.cn

## Abstract

We have synthesized a series of transition metal compounds [M(L)_2_(H_2_O)_2_] (M = Co (1), Ni (2), and Cu (3)) by using the 4-amino-1,2,4-triazole Schiff-base ligand *via* the hydrothermal methods. They are all mononuclear compounds with the octahedral geometry. Direct-current magnetic and HF-EPR measurements were combined to reveal the negative *D* values (–28.78 cm^−1^, –10.79 cm^−1^) of complexes 1 and 2, showing the easy-axis magnetic anisotropies of compounds 1 and 2. Applying a dc field of 800 Oe at 2.0 K, the slow magnetic relaxation effects were observed in compound 1, which is a remarkable feature of single-ion magnets.

## Abstract 2

We have synthesized a series of transition metal compounds [M(L)_2_(H_2_O)_2_] (M = Co (1), Ni (2), and Cu (3)) by using the 4-amino-1,2,4-triazole Schiff-base ligand *via* the hydrothermal methods.

## Graphical abstract

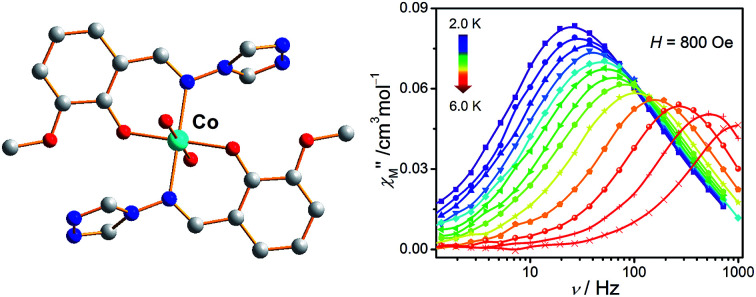

## Introduction

Single-ion magnets (SIMs), as a kind of single molecule magnets (SMMs), have been intensely studied in the past few decades due to their broad application prospects in the high-density magnetic information storage, quantum computation, and molecular spintronics.^1–3^ They also provide an ideal model for understanding the quantum phenomena in the mesoscopic world. As is known, the large ground-state spin *S* and zero-field splitting (ZFS) parameter *D* decide the relaxation energy barriers (*U*_eff_), which are treated as the reason for the slow magnetic relaxation of SMMs.^4^ We usually describe the energy barrier of transition metals as *U*_eff_ = |*D*|*S*^2^ or *U*_eff_ = (*S*^2^ − 1/4)|*D*|.^5^ The SIMs of transition metal ions and single paramagnetic lanthanide and actinide ions have been widely investigated, such as Mn(iii,iv),^6,7^ Fe(ii),^8,9^ Fe(iii),^10^ Co(i,ii),^11^ Ni(ii),^14,15^ Dy(iii)^16,17^ and Tb(iii).^18,19^ Noteworthily, the Co(ii) ion is an admirable candidate for constructing SIMs due to its large ground-state spin (*S*) and large magnetic anisotropy (*D*) on the basis of experimental and theoretical calculations. Moreover, the magnetic anisotropy of SIMs can be easily adjusted by the interactions between the ligand field splitting and the spin–orbit coupling, and the ZFS parameters are determined by the coordination structural pattern and the distortion degree of its configurations. So far, a mass of Co(ii)-based SIMs with variable coordination numbers from 2 to 8 and different coordination geometries, such as the distorted trigonal-planar, square-pyramidal, octahedral polyhedron and the like, have been investigated.^11^ It is noted that most of the Co(ii)-based SIMs with distorted octahedron geometries show the positive zero-field splitting parameter^11*b*,11*c*,11*g*,11*i*^ (*D* > 0). However, six-coordinate Co(ii)-SIMs with negative *D* values are rarely reported.^11*k*,11*f*,12,13,20^ The typical example is a chiral star-shaped Co^II^Co_3_^III^ compound (HNEt_3_)^+^(Co^II^Co_3_^III^L_6_)^−^[H_2_L = *R*-4-bromo-2-((2-hydroxy-1-phenylethylimino)methyl)phenol] reported by Gao *et al.*^11*a*^ This compound has a distorted trigonal prismatic configuration around Co^II^ ion, showing the slow magnetic relaxation behaviours with a large negative *D* value. Novikov *et al.* have also reported a negative *D* in a six-coordinate mononuclear Co(ii)-SIM with trigonal prism geometry.^11*k*^ Recently, Zhang and co-workers observed that a series of hexa-coordinate Co(ii) complexes with trigonal antiprismatic geometries exhibit the slow relaxation behaviours with the negative *D* values.^20^

As the sign of the magnetic anisotropy parameter *D* is associated with the coordinated geometries of the hexa-coordinate Co(ii)-based SIMs, it was proposed by Gomez-Coca *et al.*^11*f*^ that the six-coordinate high-spin systems (*S* = 3/2) with the twisted octahedral geometry could lead to positive ZFS parameters (*D* > 0), while trigonal prism and antiprismatic geometries are in accordance with negative ones (*D* < 0). In this context, we report a six-coordinate Co(ii) compound with the 4-amino-1,2,4-triazole Schiff-base ligand having a slightly distorted octahedral geometry (1). The compound shows a negative *D* value and the slow magnetic relaxations. As the Schiff base ligands can easily form stable compounds with most of the transition metallic ions in diverse valence states and different manners of coordination,^21^ we also chose the 4-amino-1,2,4-triazole Schiff-base ligand to synthesize a series of transition metal compounds with nickel(ii) (2) and copper(ii) (3) ions.

In our study, all of the three compounds with the octahedral geometry structure. 1 and 2 possess the negative magnetic anisotropy, and 1 shows the field-induced slow magnetic relaxation. Magnetization and HF-EPR measurements were adopted to characterize the magnetic properties of the three compounds.

## Experimental section

### General

All the reagents were commercially purchased without further purification. The IR spectra were recorded on a Nicolet 5DX spectrometer with the wavenumber in the range of 400–4000 cm^−1^ (KBr pellets). Powder X-ray diffraction (PXRD) patterns were measured on a X'Pert PRO automated diffractometer at the room temperature (Cu Kα, *λ* = 1.5406 Å). Thermogravimetric analyses (TGA) were performed in a flow of nitrogen at a heating rate of 10 °C min^−1^ using a NETZSCH TG 209 F3. Magnetic properties of polycrystalline samples were measured in the temperature range of 2–300 K and the field up to 7 T using a Quantum Design VSM SQUID magnetometer. High frequency/field electron paramagnetic resonance (HF-EPR) were measured on a locally developed instruments with pulsed magnetic fields.^22^

### Synthesis

The ligand was synthesized by methods previously reported in the literature^23,24^ (Scheme S1†). Compound 2 [Ni(L)_2_(H_2_O)_2_] were synthesized by solvothermal method at 90 °C. However, the compound 1 [Co(L)_2_(H_2_O)_2_] and compound 3 [Cu(L)_2_(H_2_O)_2_] were synthesized on the basis of the reported procedure.^24^ PXRD spectra (see the details in ESI, Fig. S1†) were used to verify the phase purity of compounds 1–3. As is seen, the experimental patterns agree well with the calculated patterns. TGA (thermogravimetric analysis) results (Fig. S2†) show that there are no guest molecules in compound 1–3. In the IR spectra, the broad peak at 3519 cm^−1^ in free ligand was due to characteristic vibrations of O–H, while it was not present in compound 1–3, suggesting the loss of H atom on the HL. The absorptions (a strong band at 1461 cm^−1^ in 1, 1464 cm^−1^ in 2, and 1463 cm^−1^ in 3) confirm the intense vibrations of C=N, which appeared at 1480 cm^−1^ in HL. The red shift of absorption peak was attributed to the coordination of the N atoms of the azomethine groups with the central metal ions.

### X-ray structural determination

The diffraction data were collected on a Bruker APEX-II CCD diffractometer with graphite-monochromatized Mo Kα radiation (*λ* = 0.071073 nm). The structures of the complexes were solved by direct methods using SHELXS-97 and refined by full-matrix least-squares on *F*^2^ using SHELXS-97. All non-hydrogen atoms were refined anisotropically. All hydrogen atoms were positioned geometrically and refined as riding. Experimental details of crystal data, data collection parameters and refinement statistics for compounds 1–3 are summarized in Table 1, while the selected bond lengths and angles are presented in Table S1.†

**Table 1:** Crystal data and structure refinements for compounds 1–3

Compound	1	2	3
Formula	C_20_H_22_CoN_8_O_6_	C_20_H_22_NiN_8_O_6_	C_20_H_22_CuN_8_O_6_
Weight	529.38	529.16	533.99
Crystal system	Monoclinic	Monoclinic	Monoclinic
Space group	*P*2_1_/*c*	*P*2_1_/*c*	*P*2_1_/*c*
*a* (Å)	9.0655 (2)	9.0895 (2)	8.7677 (2)
*b* (Å)	15.6324 (3)	15.5758 (3)	16.1464 (3)
*c* (Å)	7.8254 (2)	7.7921 (2)	7.8658 (1)
*α* (°)	90	90	90
*β* (°)	101.526 (2)	102.169 (2)	99.761 (2)
*γ* (°)	90	90	90
*V* (Å^3^)	1086.62 (4)	1078.39 (4)	1097.42 (4)
*Z*	2	2	2
*D*_c_ (g cm^−3^)	1.618	1.630	1.616
*F* (000)	546	548	550
*μ* (mm^−1^)	6.70	1.81	1.91
Reflection collected	6062	5599	6190
Unique reflection	1940	1924	1955
*R*_int_	0.031	0.031	0.028
*R*_1_ a, w*R*_2_^b^ [*I* > 2*σ*(*I*)]	0.0322, 0.0793	0.0338, 0.0868	0.0313, 0.0857
*R*_1_, w*R*_2_ (all data)	0.0391, 0.0858	0.0388, 0.0929	0.0381, 0.0922
GOF	1.094	1.074	1.055
Δ*ρ*_max_, Δ*ρ*_min_, (e Å^−3^)	0.41 and −0.47	0.23 and −0.41	0.32 and −0.32

a *R*_1_ = Σ||*F*_o_| − |*F*_c_||/Σ|*F*_o_|.

b w*R*_2_ = [Σw(*F*_o_^2^ − *F*_c_^2^)_2_/Σw(*F*_o_^2^)_2_]^1/2^.

## Results and discussion

### Structural description

**Fig. 1:**
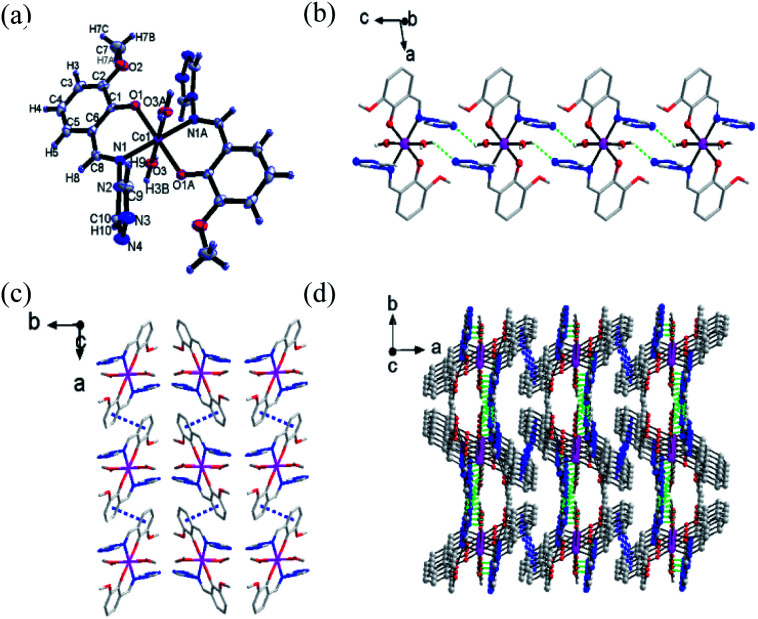
(a) The basic structure of compound 1 (thermal ellipsoids are at 50% level). (b) 1D graph formed *via* H bands of compound 1. The intermolecular H bonds are shown in green dashed lines. Other H atoms are omitted for clarity. (c) π–π stacking diagram of complex 1. The π–π interactions are shown in blue dotted lines. (d) 3D graph formed *via* H bands and π–π stacking interactions of complex 1.

Single-crystal X-ray diffraction was performed to determine the structures of compound 1–3. It revealed that 1–3 were isomorphous with similar structural parameters. Among them, the structures of 1 and 3 have already been reported.^23^ Here, we would briefly highlight 1 to discuss the following magnetic structure correlations. Their crucial crystallographic data are shown in Tables 1 and S1.† In Fig. 1, 1 is a mononuclear compound crystallized in monoclinic (*P*2_1_/n) space group. Regarding the crystallographic structure of 1, the central Co^II^ ion demonstrates a six-coordinate geometry and lies in the central location of the reverse symmetry (symmetry code A: −*x* + 2, −*y* + 1, −*z* + 1) with two ligand anions and two coordinated water molecules. Two nitrogen atoms (N1, N1A) and two oxygen atoms (O1, O1A) provided by two ligands occupy the equatorial planes, while the axial position is occupied by two oxygen atoms (O3, O3A) from the coordinated water molecules, forming a slightly twisted octahedral geometry. The axial bond angles (O3–Co–O3A) is 180.0°, the Co–O or Co–N distances are within the scope of 1.9760(14)–2.1943(15) Å. The shape calculations by the program SHAPE^25,26^ (Table. S2†) indicate the cobalt site features the distorted octahedral geometry. The elementary units are spontaneously assembled into three-dimensional supramolecular structures by way of π–π stacking interactions and hydrogen bonds. The structures of 2 and 3 are presented in Fig. S3 and S4.†

### Magnetic measurements

**Fig. 2:**
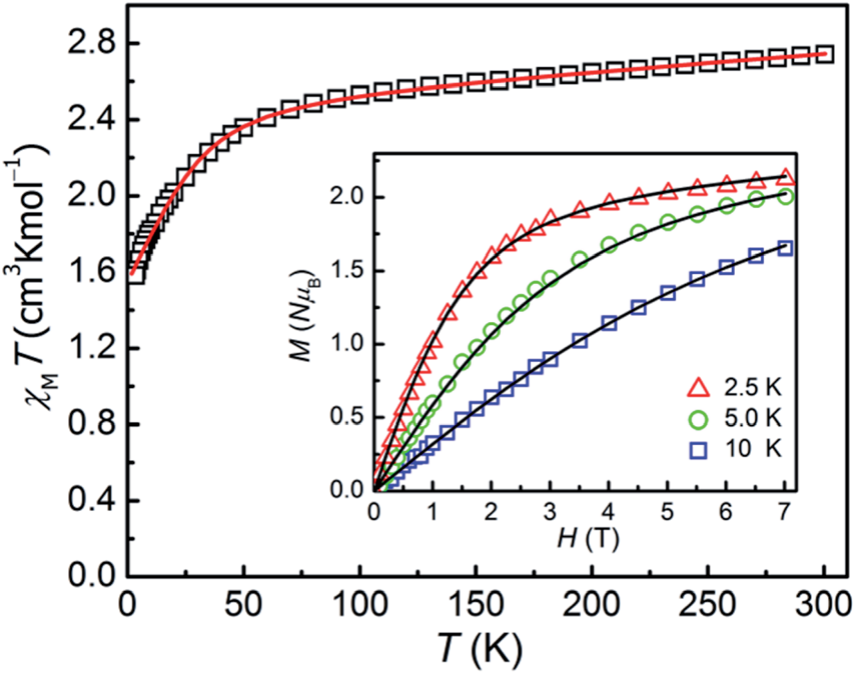
*χ*_M_ *T vs. T* plot for 1 under an applied dc field of 0.1 T. Inset: magnetization of 1 from 2.5 to 10 K. The solid lines are the best fitting results by the PHI program.

**Fig. 3:**
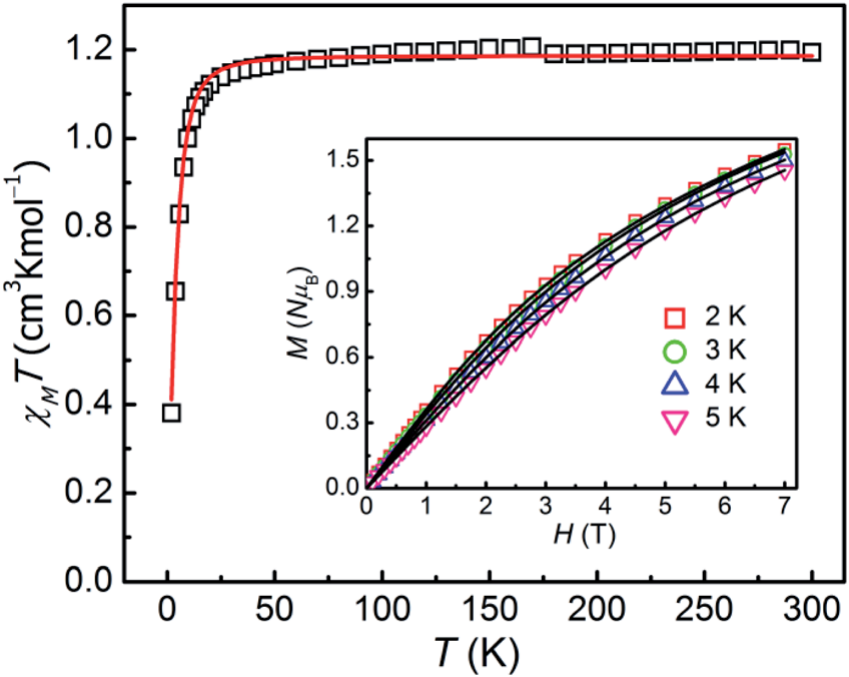
*χ*_M_ *T vs. T* plot for 2 at 1 T. Inset: magnetization of 2 within the temperature range of 2–5 K. The solid lines are the best fitting results by the PHI program.

The direct-current magnetic susceptibilities for 1–3 were measured over the temperature range of 2–300 K (Fig. 2 and 3). For compound 1, at 300 K, the resulting *χ*_M_*T* value is 2.733 cm^3^ K mol^−1^, larger than the expected value (1.875 cm^3^ K mol^−1^, *g* = 2.00) for one single high spin Co(ii) ion (*S* = 3/2), which can be due to the strong orbital contribution.^9*b*^ With the reduction of temperature, in the temperature range of 60–300 K, the *χ*_M_*T* value decreases slightly and then downs sharply to reach the minimum value of 1.60 cm^3^ K mol^−1^ at 2 K, indicating the existence of largely unquenched orbital angular momentum.^11*e*,11*h*,27,28^ The correlative field-dependent magnetization measurements were carried out within 2.5–10 K at the field range of 0–7 T (inset in Fig. 2). When the field up to 7 T, the maximum magnetization value is 2.1 *N*μ**_B_ at 2 K, which is smaller compared with the theoretical saturation value (3 *Nμ*_B_ for *S* = 3/2, *g* = 2). The unsaturated *M vs. H* plot at high field supports the existence of the magnetic anisotropy in 1.

As for 2, the *χ*_M_*T* product is measured to be 1.194 cm^3^ K mol^−1^ at 300 K, which is larger than the theoretical spin-only *χ*_M_*T* value (1.00 cm^3^ K mol^−1^, *g* = 2.00) and is expected for six-coordinate Ni(ii) ions with the momentous spin–orbit contribution. Upon cooling, the *χ*_M_*T* value decreases slowly before 30 K, and then sharply drops down to 0.38 cm^3^ K mol^−1^ at 2 K, which might be due to ZFS of the Ni(ii) ion and Zeeman effect. The field-dependence magnetization of 2 was performed with the magnetic field of 0–7 T and at temperatures of 2–5 K respectively (inset in Fig. 3). As increasing the magnetic field, the magnetization gradually increases and then reaches 1.55 *Nμ*_B_ at 7 T and 2 K, which thus is smaller than the theoretical saturation value of 2.0 *N*μ_B_ (*S* = 1, *g* = 2.0), indicating the fundamental magnetic anisotropy of 2.

As for 3, *χ*_M_*T* value is about 0.43 cm^3^ K mol^−1^ at 300 K, lower than the theoretical value (0.45 cm^3^ K mol^−1^) for one single magnetic Cu(ii) ion (*g* = 2.2).^29^ The *χ*_M_*T vs. T* plot is nearly flat between 20 and 300 K before the sharp decrease to 0.37 cm^3^ K mol^−1^ at 2 K.

To gain further insight into magnetic properties, *χ*_M_*T vs. T* and *M vs. H* curves of compound 1–3 were fitted by PHI program^30^ using the following spin Hamiltonian:

1 *Ĥ* = *gμ*_B_*BŜ* + *D*[*Ŝ*_*z*_^2^ − *S*(*S* + 1)/3] + *E*(*Ŝ*_*x*_^2^ − *Ŝ*_*y*_^2^)

where *μ*_B_, *D*, *E*, *S*, and *B* represent the Bohr magneton, axial and rhombic ZFS parameters, the spin operator, the magnetic field vector, respectively. The best-fitting parameters are presented in Table 2.

**Table 2:** Fitting parameters to the susceptibility and magnetization data of 1–3

Compound	*g*_*x*_	*g*_*y*_	*g*_*z*_	*D* (cm^−1^)	|*E*| (cm^−1^)
1	2.24(2)	2.24(2)	2.36(2)	−26.89(5)	7.55(5)
2	2.18(2)	2.17(2)	2.17(2)	−11.65(5)	2.69(5)
3	2.13(2)	2.13(2)	2.13(2)	—	—

### High-field EPR studies

It is common known that the sign of magnetic anisotropy plays a significant role in the magnetic behaviours of high-spin Co(ii) and Ni(ii) compounds. The six-coordinate Co(ii) and Ni(ii) compounds with the octahedral geometric structure have been investigated to show fine both positive and negative easy-axis magnetic anisotropies.^11*b*,31–36^

**Fig. 4:**
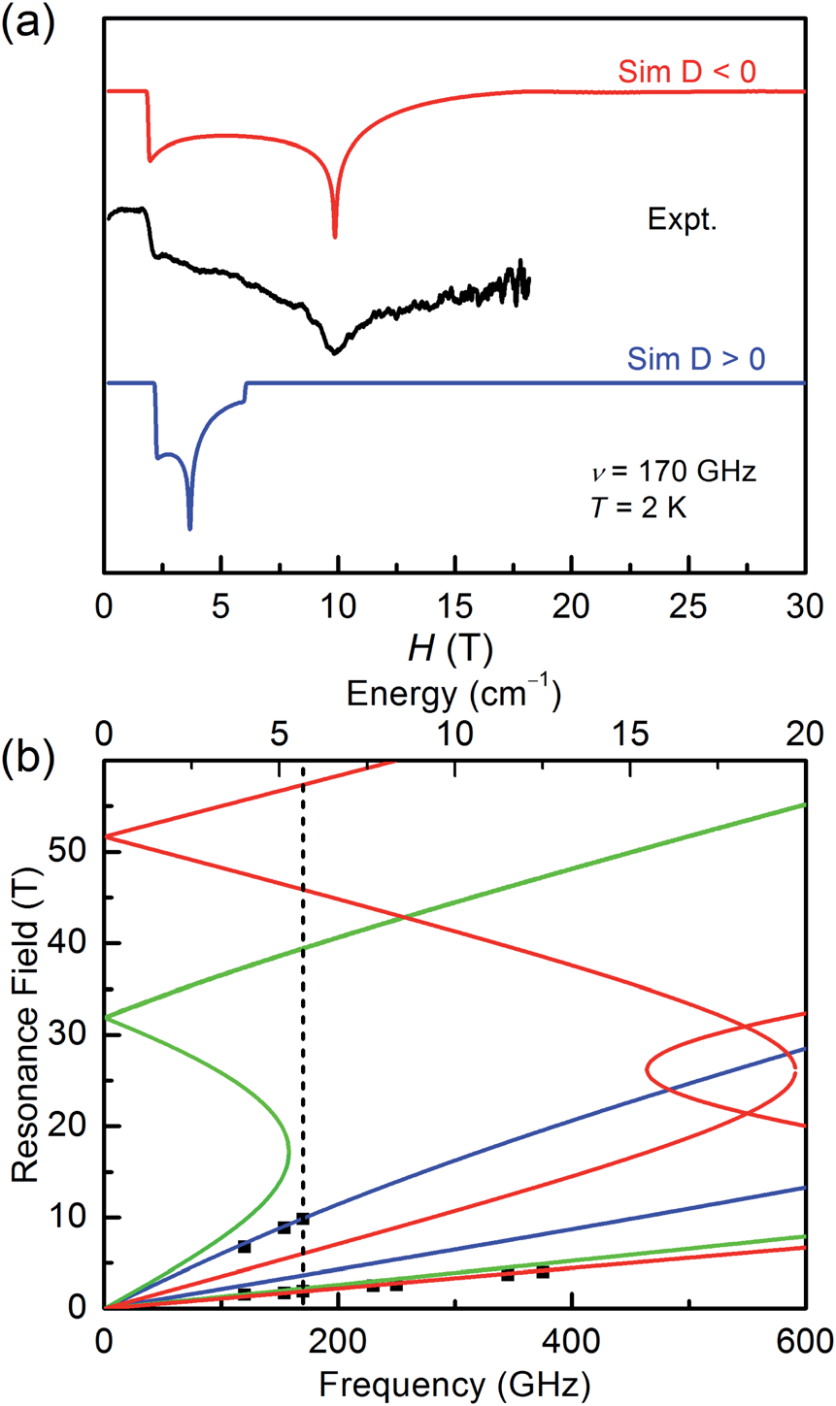
(a) The HF-EPR spectrum of 1 with its simulations at 2 K and 170 GHz. The blue trace represents the simulation with a positive *D* value, and the red race represents the simulation with a negative *D* value. (b) Plots of HF-EPR resonances *vs.* frequency for 1 at 2 K. Squares are the experimental data, and blue, green, and red lines are the calculated lines using the spin-Hamiltonian parameters for the magnetic fields parallel to the molecular axes *X*, *Y*, and *Z*, respectively. The vertical dashed line is the frequency (170 GHz) at which the spectrum was taken and simulated.

**Fig. 5:**
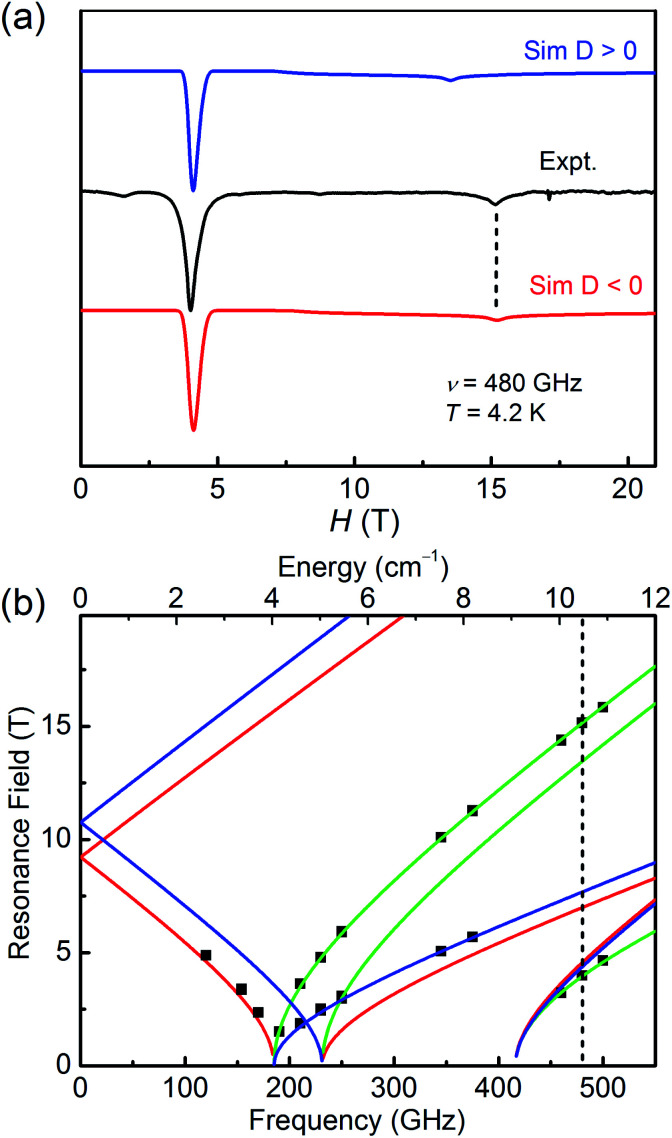
(a) The HF-EPR spectrum of 2 with its simulation at 4.2 K and 480 GHz. The blue trace represents the simulation with a positive *D* value, and the red race represents the simulation with a negative *D* value. (b) Complete resonance field *vs.* frequency dependence of EPR transitions in 2. The squares are experimental points at specific frequencies while the fitting results using the spin-Hamiltonian parameters are represented by lines. The vertical dotted line is the frequency (480 GHz) at which the spectrum was taken and simulated.

The high field/frequency electron paramagnetic resonance (HF-EPR) measurements were carried out to confirm the sign of *D* because the sign of *D* obtained from the magnetic data is not always reliable. The polycrystalline powders of 1–3 were measured in the frequency range of 60–500 GHz (Fig. 4, 5, and S5†). As the amplitude of *D* for 1 (∼28.78 cm^−1^) is much larger than the available microwave quantum energy (500 GHz ∼ 16.7 cm^−1^), no transition between Kramers doublets *M*_S_ = ±1/2 and *M*_S_ = ±3/2 can be seen,^11*j*,37,38^ so the HF-EPR spectra of 1 were simulated base on the amplitude of *D* and the *g* values obtained from SQUID measurements and adjusting the ZFS parameter *E*, yielding |*E*| = 4.78 cm^−1^ (Fig. 4b). As shown in Fig. 4a, two simulations were done with different signs of *D*, showing that the negative *D* value are well in accord to the experimental data. But beyond that, there are only two peaks in the spectra for 1, which is typical for high-spin 3/2 Co(ii) systems with large negative *D* values due to the limit of magnetic field.^39^

As for 2, the well-resolved powder-pattern spectrum at 480 GHz of a triplet state (*S* = 1) were received as shown in Fig. 5a (black trace). Simulations with positive (blue trace) and negative (red trace) *D* values evidence that the *D* value is negative, indicating the easy-axis magnetic anisotropy of 2. The correlative parameters are given as follows: *D* = −10.79(5) cm^−1^, *E* = 3.08(5) cm^−1^, *g*_*x*_ = *g*_*y*_ = 2.15(2), and *g*_*z*_ = 2.05(2). These parameters are close to the obtained values from magnetic studies above (Table 1) and the values of hexa-coordinate Ni(ii) compounds in literature.^40–45^ The entire resonance field *vs.* frequency plot of 2 is demonstrated in Fig. 5b. Resonances drawn in this way forms characteristic branches, which are labelled according to Wasserman's terminology.^46^ The calculation lines through these points are based on the combination of automatic nonlinear least square fitting which uses artificial judgment to eliminate the physical unreasonable results.

The HF-EPR spectrum of 3 was recorded at 154 GHz and 4.2 K (Fig. S5†). Three main peaks were observed which correspond to the anisotropic *g* values of the Cu(ii) ion with *S* = 1/2. The simulation to the spectrum results in the *g* values as *g*_*x*_ = 2.03(2), *g*_*y*_ = 2.07(2), and *g*_*z*_ = 2.27(2) with *g*_iso_ = 2.13, which agrees well with the magnetic studies (Table 1).

### Dynamic (ac) magnetic properties of 1

**Fig. 6:**
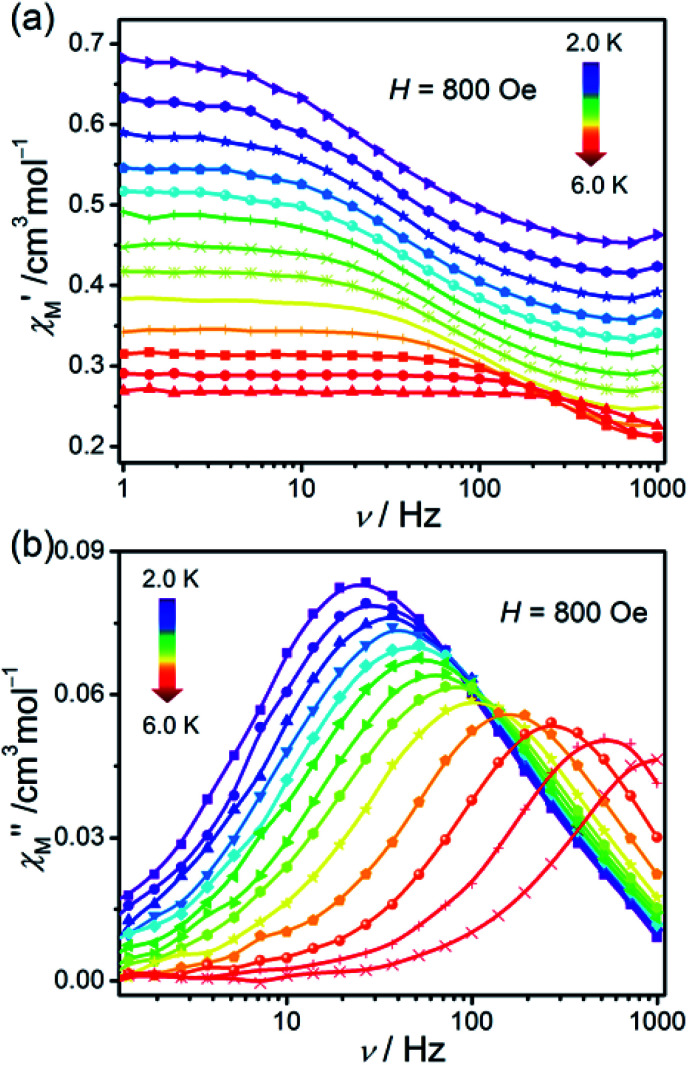
Frequency dependence of ac susceptibilities for the compound 1 performed under 800 Oe dc field from 2 to 6 K. The solid lines are guides to the eyes.

In order to investigate the dynamic magnetic behaviours at low temperatures, ac susceptibilities were measured at 2–6 K for 1. When no dc field was applied, no out-of-phase ac susceptibility (*χ*′′) signal could be observed at 2 K, which can be attributed to the existence of the magnetized quantum tunnelling (QTM). With the external magnetic field increases, the *χ*′′ signal could be seen, suggesting that the QTM effect could be suppressed by the applied dc field. At 2.0 K and 1.0–996.5 Hz, the variable dc fields were performed to measure the out-of-phase ac susceptibilities for 1 to find an optimum applied magnetic field which can suppress the QTM phenomenon (Fig. S6†). Consequently, an optimum field of 800 Oe (at which the *χ*′′ signals in a lower frequency range and with enough amplitude) was applied to detect the frequency and temperature-dependent ac susceptibilities within 2–6 K (Fig. 6 and S7†), and the field-induced slow magnetic relaxation of 1 was observed.

**Fig. 7:**
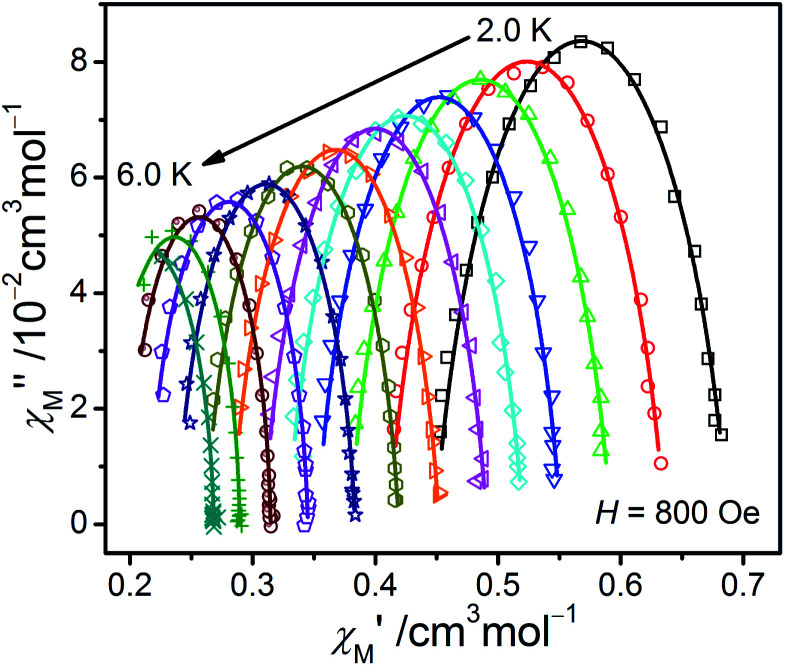
The Cole–Cole plots obtained at 2.0–6.0 K and 800 Oe for 1. The solid lines best conform to the experimental data.

From the ac data collected above, the Cole–Cole diagrams were charted as a form of *χ*_M_′′ *vs. χ*_M_′ at 2–6 K and 800 Oe dc field (Fig. 7). Fitting was performed by using the generalized Debye model as following (eqn (2)):2
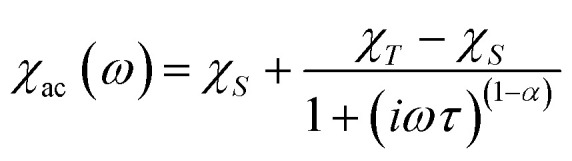
where *χ*_T_ and *χ*_S_ represent the isothermal and adiabatic susceptibilities, respectively, *ω* represents the angular frequency, *τ* represents the relaxation time, and *α* indicates the deviation from a pure Debye model.^47,48^ The correlative fitting parameters are listed in Table S3.† The parameter *α* is from 0.025 to 0.22, which indicates that the distribution of relaxation time of compound 1 is small. Fitting the values of relaxation time within 2.0–10.0 K in the light of the Arrhenius law *τ*^−1^ = *τ*_0_^−1^exp(−*U*_eff_/*kT*) results in *U*_eff_ = 43.70 K and *τ*_*0*_ = 1.12 × 10^−7^ s (Fig. S8†). Comparing with the energy gap assessed from the value of |2*D*| (57.56 cm^−1^), the obtained *U*_eff_ is rather small, and the evident curvature in the Arrhenius plot of 1 at low temperature manifests that there could exist multiple relaxation pathways, including non-negligible Raman or Raman-like, direct, and Orbach mechanisms. It is worth noting that the contributions of a Raman process cannot be neglected and a direct one-phonon will make some contributions at lower temperatures in terms of most of the Co(ii)-based SIMs reported.^11*g*,11*j*,49^ Therefore, a model including two possible mechanisms were applied to analyse this relaxation behaviour by eqn (3):^29,49*a*^

3 *τ*^−1^ = *CT*^*n*^ + *AT*

where the two terms in eqn (3) relate to the contributions of Raman or Raman-like and Direct mechanisms. The best fit of experimental data is shown in Fig. S9† with the parameters *n* = 7.97, *C* = 0.0037 K^−7.97^ S^−1^, and *A* = 99.12 K^−1^ S^−1^. Compared with the anticipated value 9 of the Raman process of Kramers ions, the *n* obtained value of 7.97 is smaller, stating possibly a photoacoustic Raman process refer to virtual states, in which both of the optical and acoustic phonons are all considered.^49,50^ We can conclude that the slow relaxation behaviour is the result of synergistic effect of Raman-like and Direct processes.

No ac signal was observed on 2 and 3 under the dc fields of 0–0.1 T were applied (Fig. S10†), indicating that 2 and 3 don't possess the properties of SIM or spin-phonon trapping properties.^51–53^

## Conclusions

To sum up, we have successfully synthesized three 3d transition metal compounds based on 4-amino-1,2,4-triazole Schiff-base ligand *via* hydrothermal method and their structures were further characterized. All of 1, 2, and 3 are mononuclear complexes with the octahedral geometry. Magnetic measurements and HF-EPR studies manifest the easy-axis anisotropy with negative *D* parameters in 1 and 2, which might be due to the elongated octahedron of metal geometry. Ac magnetic susceptibility measurements confirm that 1 exhibit the typical field-induced slow magnetic relaxation behaviours, while no slow magnetic relaxations were observed in 2 and 3. We have added one more example to the six-coordinate cobalt(ii) compounds showing the field-induced SIM behaviours with the negative magnetic anisotropy, which was rarely reported. Its dynamic magnetic behaviours could be explained *via* a Raman-like process in high-temperature zone while the relaxation occurs *via* a direct process within the lower temperature range.

## Conflicts of interest

There are no conflicts to declare.

## Supplementary Material

RA-010-C9RA10851C-s001

RA-010-C9RA10851C-s002
