# Supplementary material for: Synthesis, crystal structures, HF-EPR, and magnetic properties of six-coordinate transition metal (Co, Ni, and Cu) compounds with a 4-amino-1,2,4-triazole Schiff-base ligand
Source: RSC Adv. 2020 Mar 31;10(22):12833–40. doi: 10.1039/c9ra10851c (PMC9051221; doi:10.1039/c9ra10851c)
Supplement: RA-010-C9RA10851C-s001 [file RA-010-C9RA10851C-s001.pdf]

Electronic Supplementary Material (ESI) for RSC Advances.
This journal is © The Royal Society of Chemistry 2020

Supporting Information for

Synthesis, Crystal structures, HF-EPR, and Magnetic properties of Six-coordinate Transition Metal (Co, Ni, and Cu) Compounds with 4-Amino-1,2,4-triazole Schiff-base Ligand

Ya-Jie Zhang,[a,⊥] Lei Yin,[a,⊥] Jing Li,[b] Zhao-Bo Hu,[b] Zhong-Wen Ouyang,[a] You Song[*,b] and Zhenxing Wang,[*,a]

[a]Wuhan National High Magnetic Field Center & School of Physics, Huazhong University of Science and Technology, Wuhan, Hubei, 430074, China.

[b]State Key Laboratory of Coordination Chemistry, School of Chemistry and Chemical Engineering, Nanjing University, Nanjing 210023, P. R. China.

E-mails: zxwang@hust.edu.cn (ZW), yousong@nju.edu.cn (YS)

[⊥] These authors contributed equally.

Table of contents

1. Experimental section
2. Single crystal X-ray characterizations
3. Powder X-ray characterizations
4. Thermogravimetric analysis
5. Structures
6. Magnetic studies
7. References

1. Experimental section

Synthesis of 2-hydroxy-3-methoxybenzylidene-4H-1,2,4-triazol-4-amine (HL). An alcoholic solution of 4-amino-4*H*-1,2,4-triazole (0.842 g,10 mmol) was slowly added to a solution of *o*-Vanillin (1.522 g,10 mmol) in 15 ml of EtOH. The reaction mixture was refluxed for 4 h at 80 °C. A pale yellow colored solid was obtained when the reaction mixture was cooled to ambient temperature and which was then washed with hot EtOH. After sufficient drying, the pure ligand was obtained[1] with a yield of 65% (scheme S1).

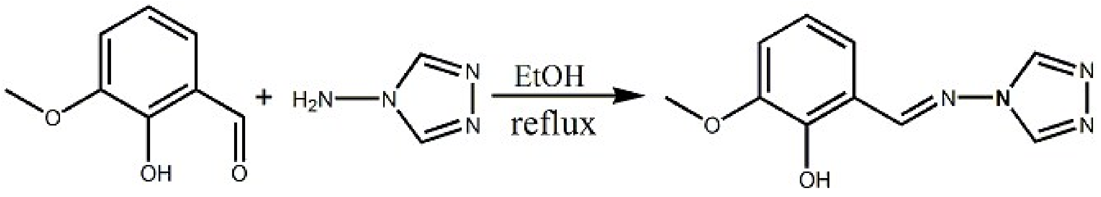

Scheme S1. Synthesis of ligand HL.

Synthesis of [Co(L)$_2$(H$_2$O)$_2$] (1). A mixture of Co(CH$_3$COO)$_2$·4H$_2$O (12.5 mg, 0.05 mmol) and HL (22 mg, 0.1 mmol) were dissolved in 5 ml CH$_3$OH/H$_2$O (1:4, v:v). The mixture was sealed into a 10 ml hydrothermal reactor and heated to 90 °C for 72 h under auto genous pressure. Then the system was cooled to ambient temperature at the rate of 3 °C/h. The pale red block crystals were formed [2] with a yield of ca. 62.3% (based on Co), washed with mother liquor and dried in air at room temperature, which were suitable for X-ray structural determination. FTIR (KBr pellet, cm^{-1}): 3437(br), 3086(m), 1685(m), 1587(s), 1461(s), 1343(m), 1237(s), 1053(s), 858(m), 715(m), 644(m), 561(s).

Synthesis of [Ni(L)$_2$(H$_2$O)$_2$] (2). The complex **2** was synthesized by a similar method of **1** except that Co(CH$_3$COO)$_2$·4H$_2$O was replaced by Ni (CH$_3$COO)$_2$·4H$_2$O (12.4 mg, 0.05 mmol). The green block crystals were obtained with a yield of ca. 57.1% (based on Ni). FTIR (KBr pellet, cm^{-1}): 3446(br), 3087(m), 1682(m), 1589(s), 1464(s), 1348(m), 1216(s), 1053(s), 860(m), 715(m), 646(m), 560(s).

Synthesis of [Cu(L)$_2$(H$_2$O)$_2$] (3). A mixture of Cu(CH$_3$COO)$_2$·H$_2$O (40 mg, 0.2 mmol) and HL (44 mg, 0.2 mmol) were dissolved in 5 ml DMF, the dark green solution was stirred for 20 min and transferred into a 15 ml tube, and then 5 ml CH$_3$CN was slowly added into this solution. After undisturbed for three days, then dark green block crystals were formed with a yield of ca. 21.5% (based on Cu). FTIR (KBr pellet, cm^{-1}): 3451(br), 3088(m), 1674(m), 1585(s), 1463(s), 1342(m), 1220(s), 1054(s), 856(m), 719(m), 648(m), 568(s).

2. Single crystal X-ray characterizations

Table S1. Selected bond lengths (Å) and angles (°) for compounds **1**−**3**.

Compound **1**

Co(1)-O(1)A	1.9760 (14)	Co(1)-O(3)	2.1943 (15)
Co(1)-O(1)	1.9760 (14)	Co(1)-N(1)A	2.0955 (17)
Co(1)-O(3)A	2.1943 (15)	Co(1)-N(1)	2.0955 (17)
O(1)A-Co(1)-O(1)	180.0	O(1)A-Co(1)-O(3)	90.26 (6)
O(1)-Co(1)-O(3)	89.74 (6)	O(1)A-Co(1)-O(3)A	89.74 (6)
O(1)-Co(1)-O(3)A	90.26 (6)	O(1)A-Co(1)-N(1)A	87.21 (6)
O(1)A-Co(1)-N(1)	92.79 (6)	O(1)-Co(1)-N(1)	87.21 (6)
O(1)-Co(1)-N(1)A	92.79 (6)	O(3)A-Co(1)-O(3)	180.0
N(1)-Co(1)-O(3)	85.80 (7)	N(1)-Co(1)-O(3)A	94.20 (7)
N(1)A-Co(1)-O(3)	94.20 (7)	N(1)A-Co(1)-O(3)A	85.80 (7)
N(1)A-Co(1)-N(1)	180.0	C(1)-O(1)-Co(1)	126.76 (12)
N(2)-N(1)-Co(1)	120.31 (12)	C(8)-N(1)-Co(1)	124.35 (14)

Symmetry code: A) -*x*+2, -*y*+1, -*z*+1.

Compound **2**

Ni(1)-O(2)	1.9743 (14)	Ni(1)-O(2)A	1.9743 (14)
Ni(1)-O(3)A	2.1404 (15)	Ni(1)-O(3)	2.1404 (15)
Ni(1)-N(1)A	2.0571 (16)	Ni(1)-N(1)	2.0571 (16)
O(2)-Ni(1)-O(2)A	180.00 (5)	O(2)-Ni(1)-O(3)	90.48 (6)
O(2)A-Ni(1)-O(3)	89.52 (6)	O(2)-Ni(1)-O(3)A	89.52 (6)
O(2)A-Ni(1)-O(3)A	90.48 (6)	O(2)-Ni(1)-N(1)A	92.12 (6)
O(2)-Ni(1)-N(1)	87.88 (6)	O(2)A-Ni(1)-N(1)	92.12 (6)
O(2)A-Ni(1)-N(1)A	87.88 (6)	O(3)A-Ni(1)-O(3)	180.0
N(1)-Ni(1)-O(3)	86.83 (7)	N(1)-Ni(1)-O(3)A	93.17 (7)
N(1)A-Ni(1)-O(3)	93.18 (7)	N(1)A-Ni(1)-O(3)A	86.82 (7)
N(1)A-Ni(1)-N(1)	180.0	C(6)-O(2)-Ni(1)	126.41 (12)
N(2)-N(1)-Ni(1)	120.02 (12)	C(8)-N(1)-Ni(1)	124.53 (14)

Symmetry code: A) -*x*, -*y*+1, -*z*+1.

Compound **3**

Cu(1)-O(2)	1.9085 (13)	Cu(1)-O(2)A	1.9085 (13)
Cu(1)-O(3)A	2.4792(17)	Cu(1)-O(3)	2.4792(17)
Cu(1)-N(1)	2.0004 (16)	Cu(1)-N(1)A	2.0004 (16)
O(2)-Cu(1)-O(2)A	180.0	O(2)-Cu(1)-O(3)	91.961(58)
O(2)A-Cu(1)-O(3)	88.039(58)	O(2)-Cu(1)-O(3)A	88.039(58)
O(2)A-Cu(1)-O(3)A	91.961(58)	O(2)-Cu(1)-N(1)A	90.36 (6)
O(2)-Cu(1)-N(1)	89.64 (6)	O(2)A-Cu(1)-N(1)	90.36 (6)
O(2)A-Cu(1)-N(1)A	89.64 (6)	O(3)A-Cu(1)-O(3)	180.0
N(1)-Cu(1)-O(3)	94.189(59)	N(1)-Cu(1)-O(3)A	85.811(59)
N(1)A-Cu(1)-O(3)	85.811(59)	N(1)A-Cu(1)-O(3)A	94.189(59)
N(1)A-Cu(1)-N(1)	180.00 (9)	C(6)-O(2)-Cu(1)	126.08 (12)
N(2)-N(1)-Cu(1)	120.69 (12)	C(8)-N(1)-Cu(1)	124.55 (14)

Symmetry code: A) -x, -y+1, -z+1.

Table S2. Deviation parameters calculated by SHAPE from each ideal polyhedron for **Co1**, **Ni1**, **Cu1** in compound **1**−**3**. The best matches are shown in red.

Geometry	Symmetry	Co1	Ni1	Cu1
HP-6	D_{6h}	30.830	31.143	31.668
PPY-6	C_{5v}	28.595	28.877	28.788
**OC-6**	$\mathbf{O_h}$	**0.312**	**0.185**	**1.473**
TPR-6	D_{3h}	16.142	16.321	17.166
JPPY-6	C_{5v}	31.545	31.967	31.266

HP-6 = Hexagon, PPY-6 = Pentagonal pyramid, OC-6 = Octahedron, TPR-6 = Trigonal prism, JPPY-6 = Johnson pentagonal pyramid (J2).

3. Powder X-ray characterizations

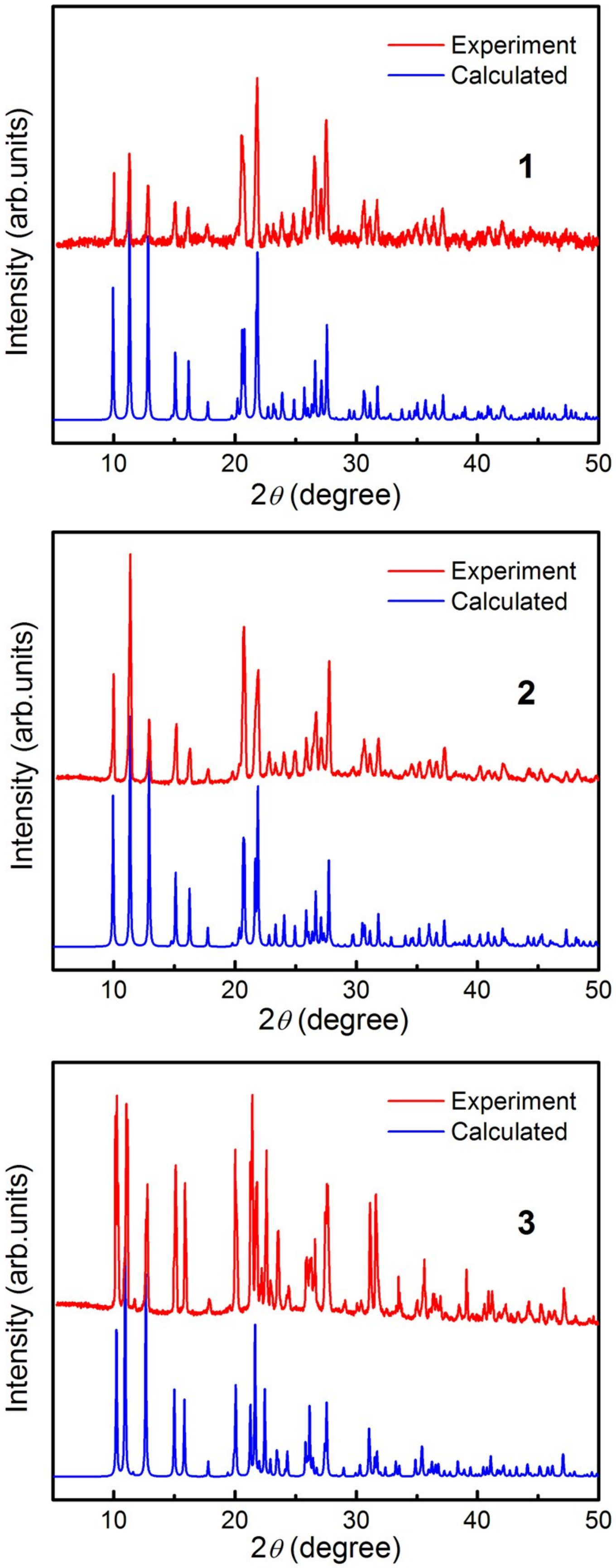

Figure S1. The calculated (blue) and the experimental (red) XRD powder patterns of **1–3**.

4. Thermogravimetric analysis

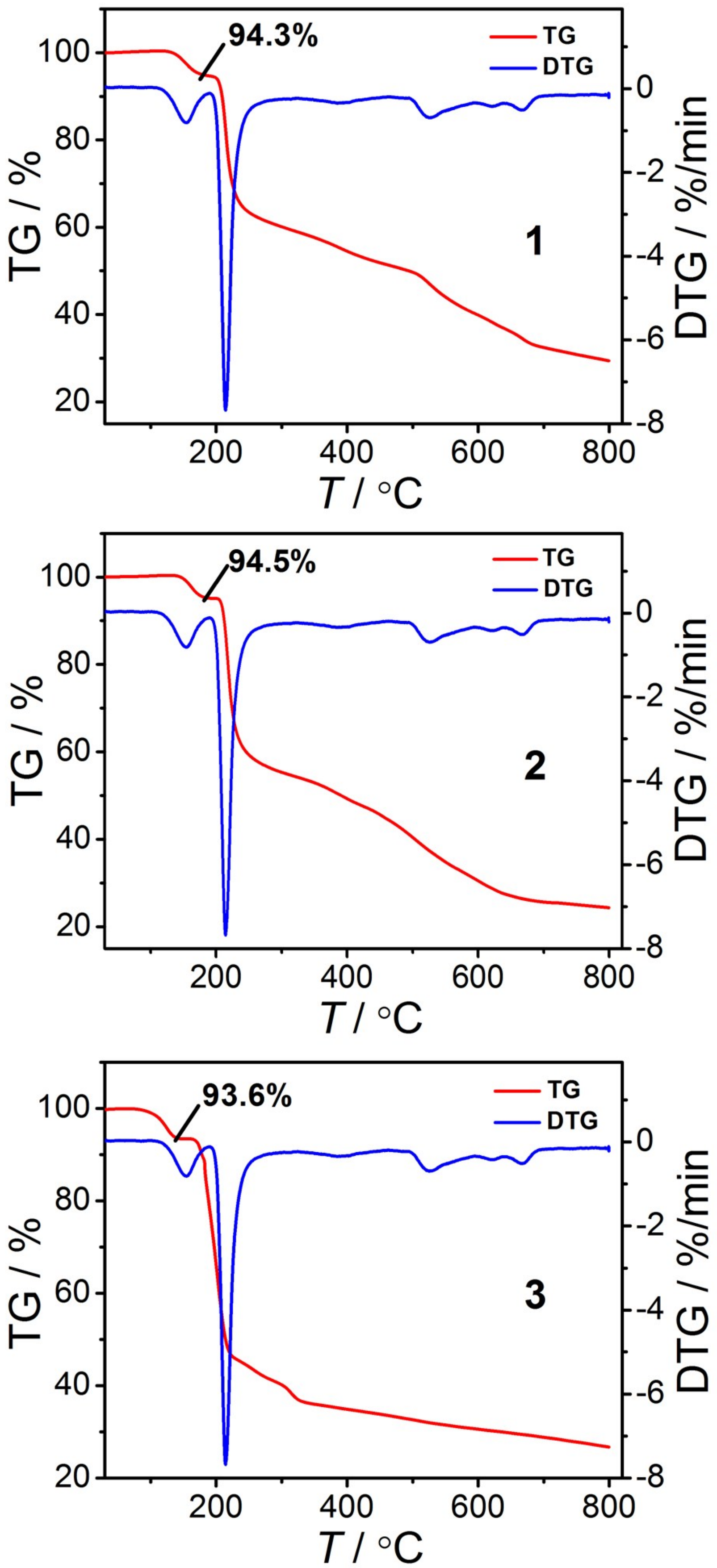

Figure S2. The TGA plots of **1–3.**

5. **Structures**

(a)

(b)

(c)

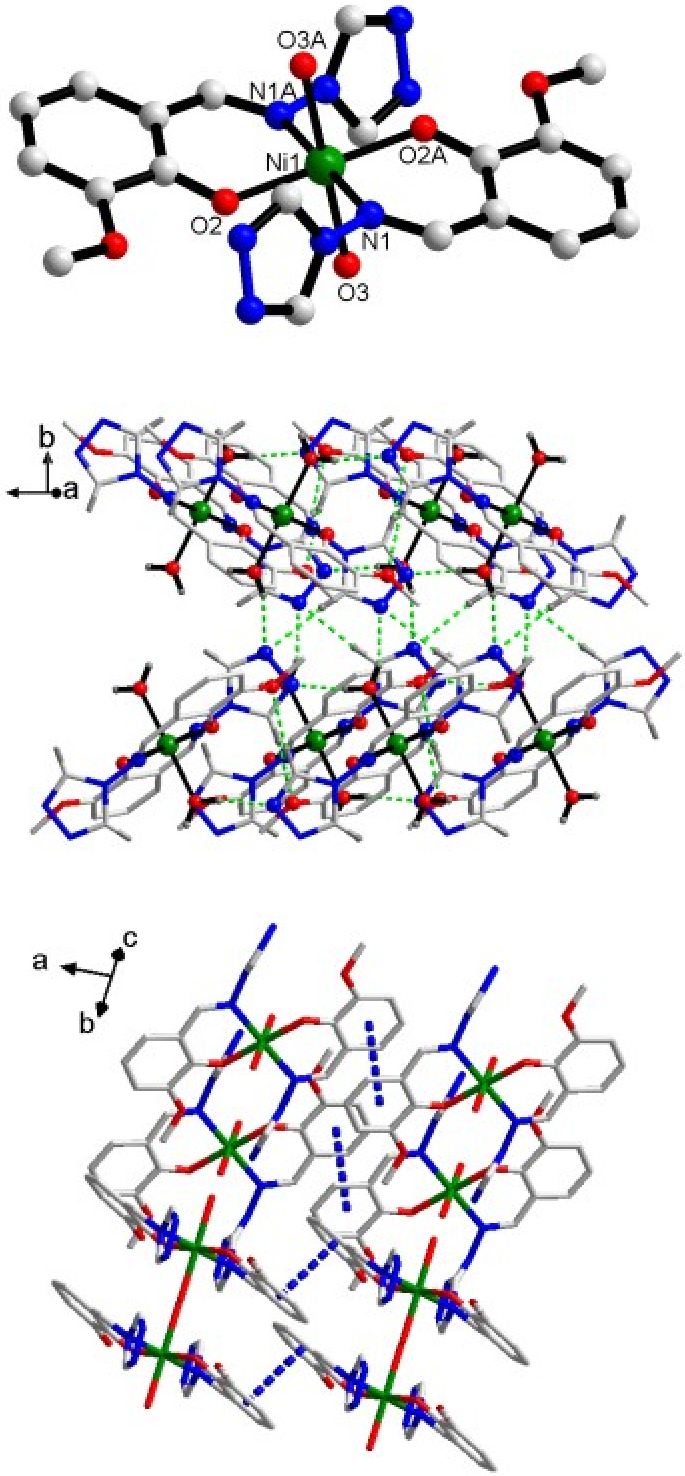

Figure S3. (a) The coordination environment of Ni(II) ion in **2**. (b) The 3D layer structure of **2**. The green dotted line represents inter-molecular H bonds. (c) π–π packing diagram of three-dimensional supramolecular structure of **2** .Hydrogen atoms are omitted for clarity.

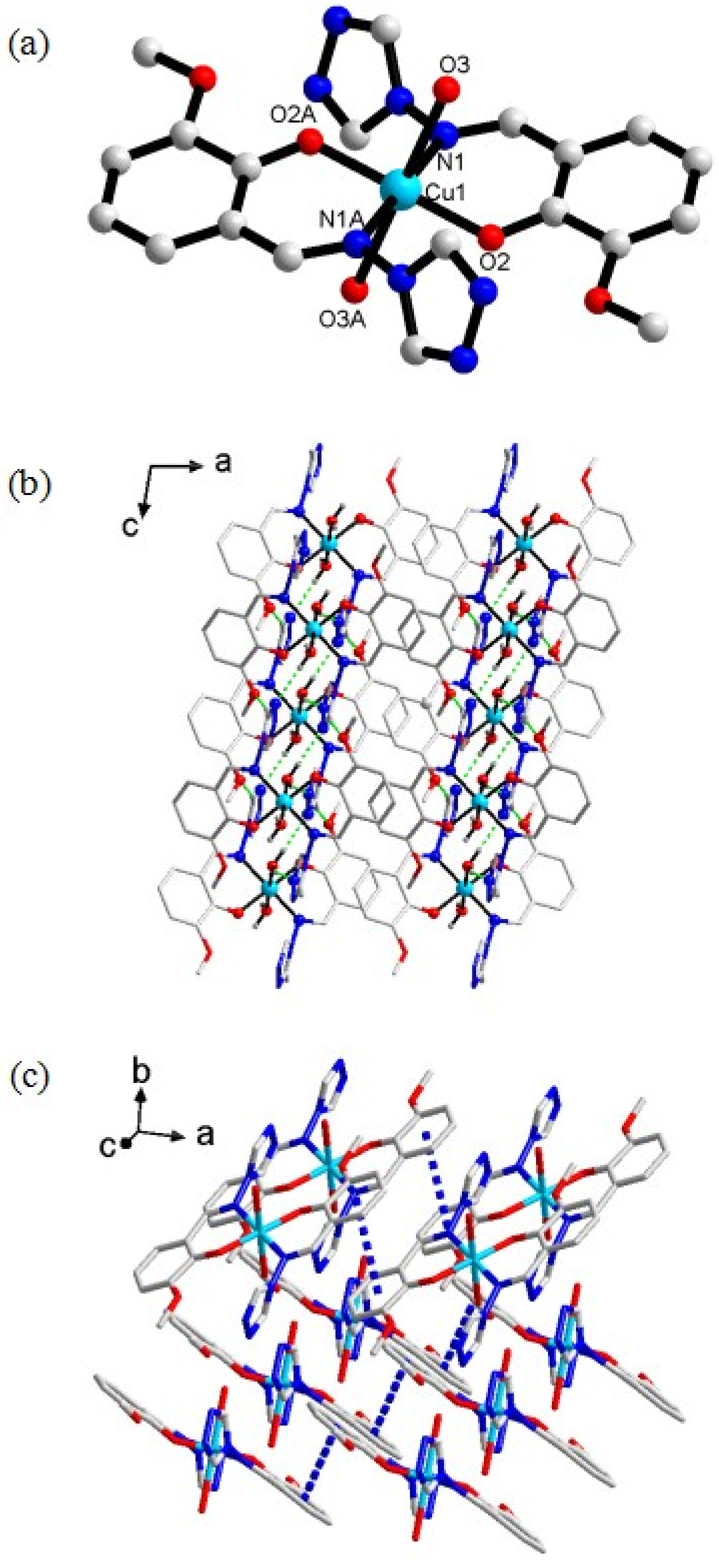

Figure S4. (a) The coordination environment of Cu(II) ion in **3**. (b) The 3D layer structure of **3**. The green dotted line represents inter-molecular H bonds. (c) π–π packing diagram of 3D supramolecular structure of **3**. Hydrogen atoms are omitted for clarity.

6. Magnetic studies

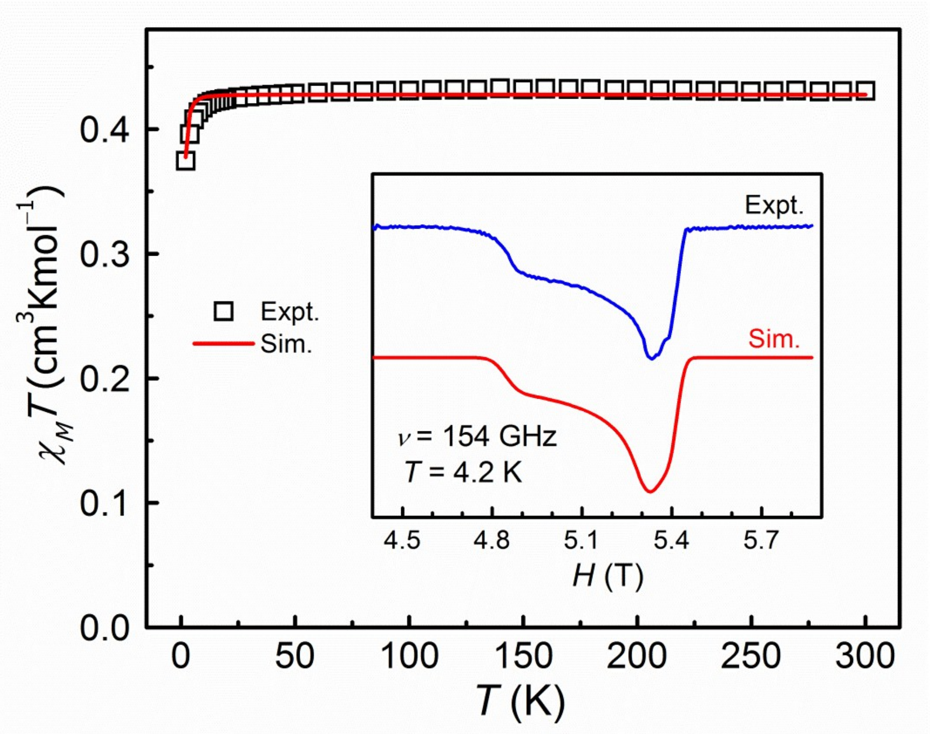

Figure S5. Variable-temperature *dc* susceptibilities of compound **3** under an applied dc field of 1 T. The red line is the best fit with the *PHI* program. Inset: HF-EPR spectrum of **3** with its simulation at 154 GHz and 4.2 K.

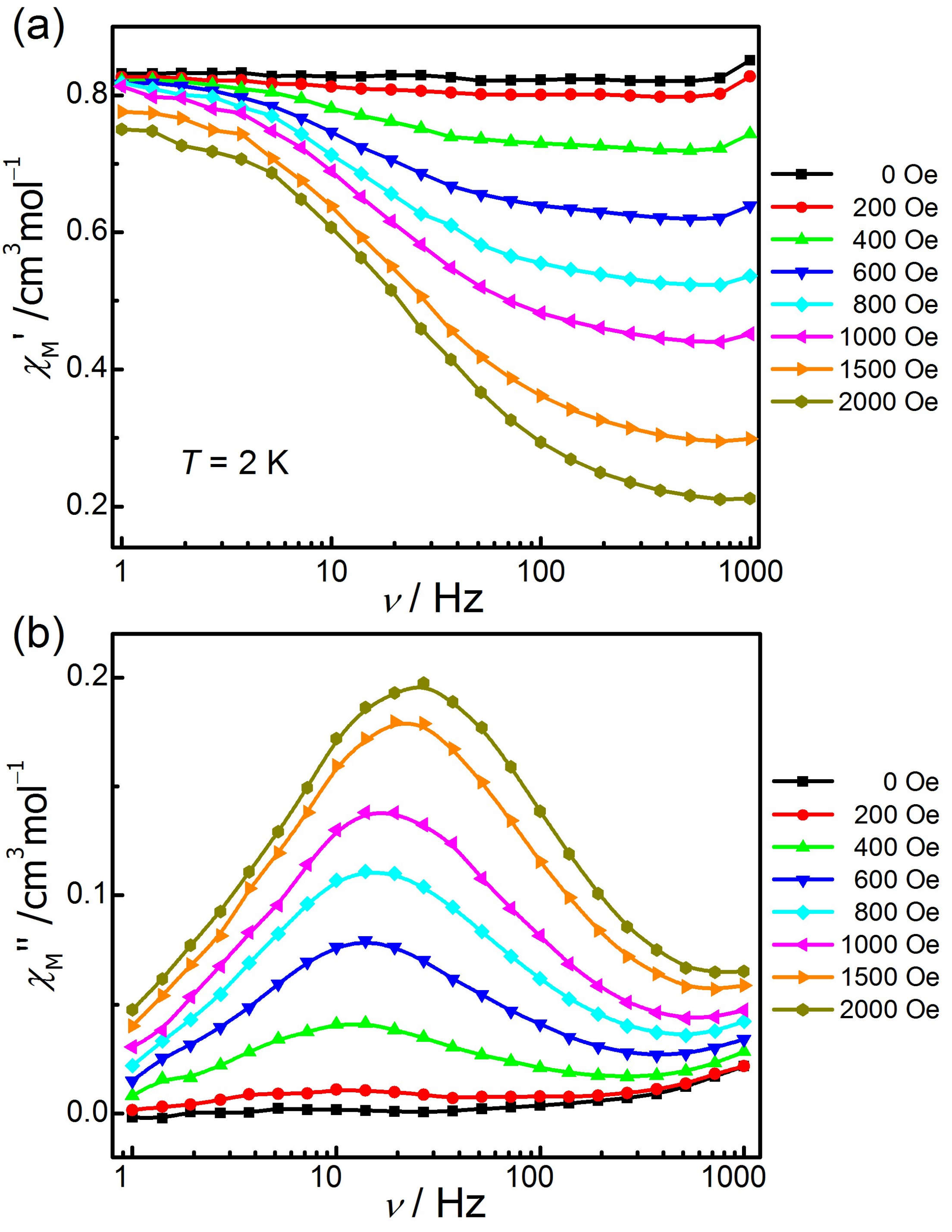

Figure S6. Frequency-dependent out of phase (χ_M'') *ac* susceptibility at 2.0 K for **1** under the different applied *dc* fields within 0–2000 Oe.

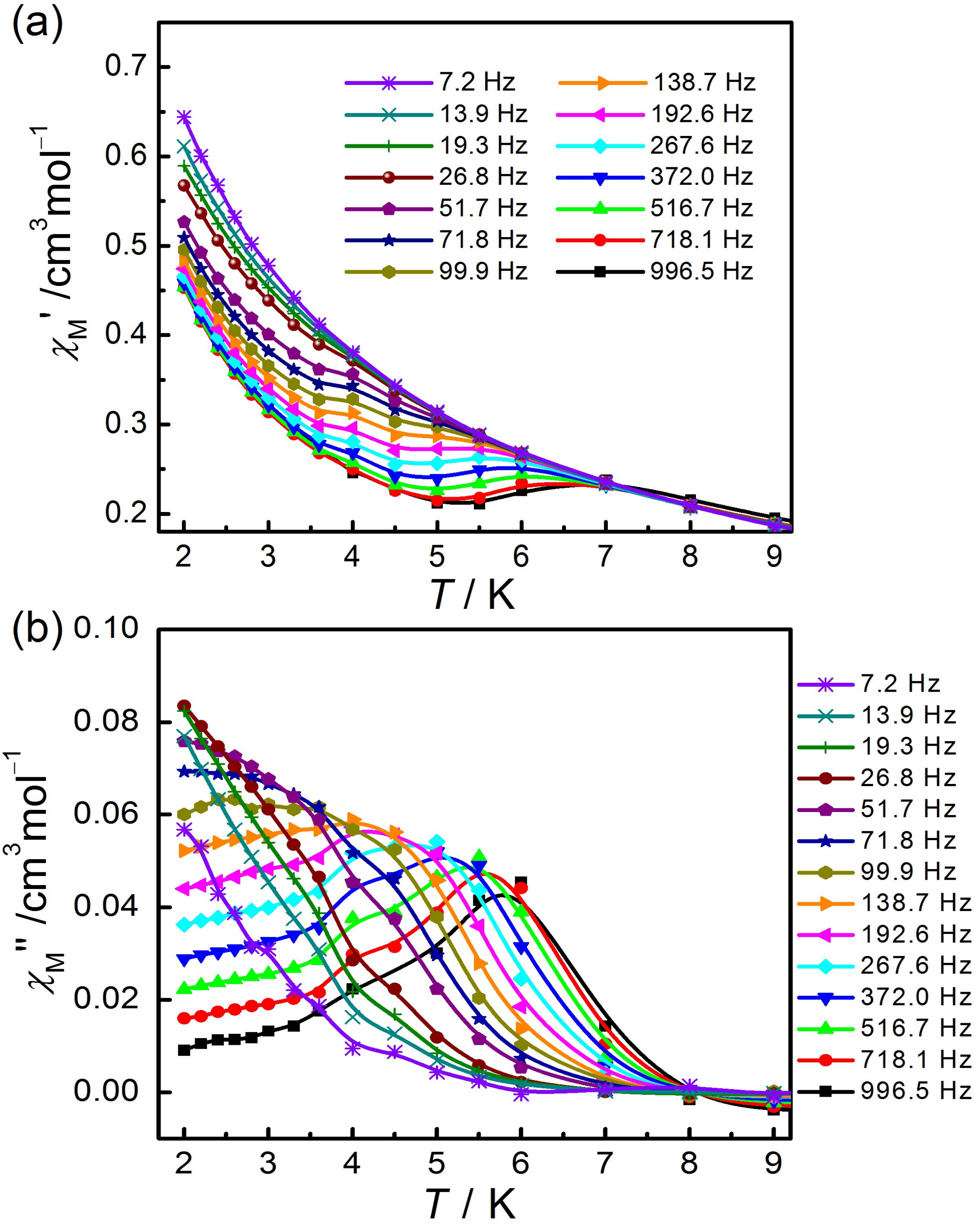

Figure S7. (a) Temperature dependence of in-of-phase (χ_M') and (b) out-of-phase ac susceptibilities (χ_M'') at variable *ac* frequencies under a *dc* magnetic field of 800 Oe for **1**. The solid lines are for eye guide.

Table S3. The best fits for **1** under 800 Oe *dc* field by a generalized Debye model.

T / K	τ /s	α	χ_s	χ_t
2.0	0.00575	0.21792	0.44856	0.68763
2.2	0.00488	0.20789	0.41081	0.63607
2.4	0.00424	0.19747	0.37912	0.59195
2.6	0.00368	0.17912	0.35252	0.55097
2.8	0.00332	0.18559	0.32849	0.52032
3.0	0.00295	0.17309	0.30869	0.49077
3.3	0.00243	0.15971	0.2832	0.45188
3.6	0.00202	0.14291	0.26199	0.41899
4.0	0.00153	0.12429	0.23846	0.38343
4.5	9.9515E-4	0.08344	0.21602	0.34498
5.0	5.89917E-4	0.04731	0.19818	0.31451
5.5	3.17554E-4	0.03118	0.18318	0.28924
6.0	1.35949E-4	0.06391	0.16201	0.26834
7.0	8.33473E-6	0.04437	9.08829E-15	0.23404
8.0	9.08843E-21	0.04974	1.12871E-14	0.20837
9.0	1.33141E-20	0.04333	9.34835E-15	0.1886
10.0	1.70401E-20	0.02516	1.30093E-14	0.17205

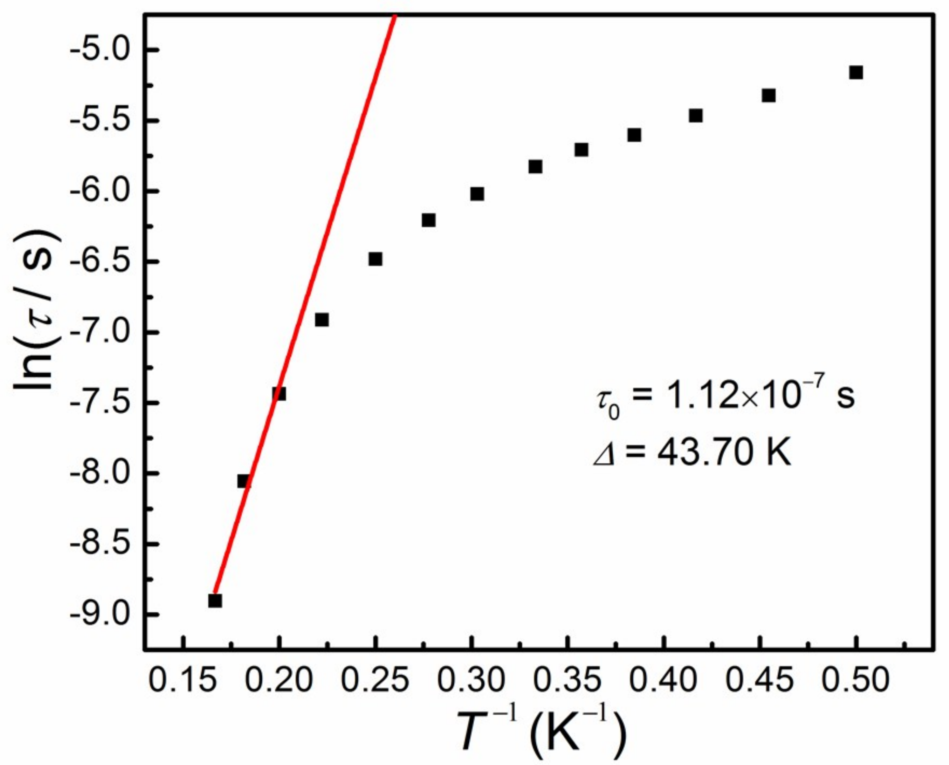

Figure S8. Relaxation time of the magnetization ln(τ) versus T^{-1} plot for **1**. The red solid line represents Arrhenius fit.

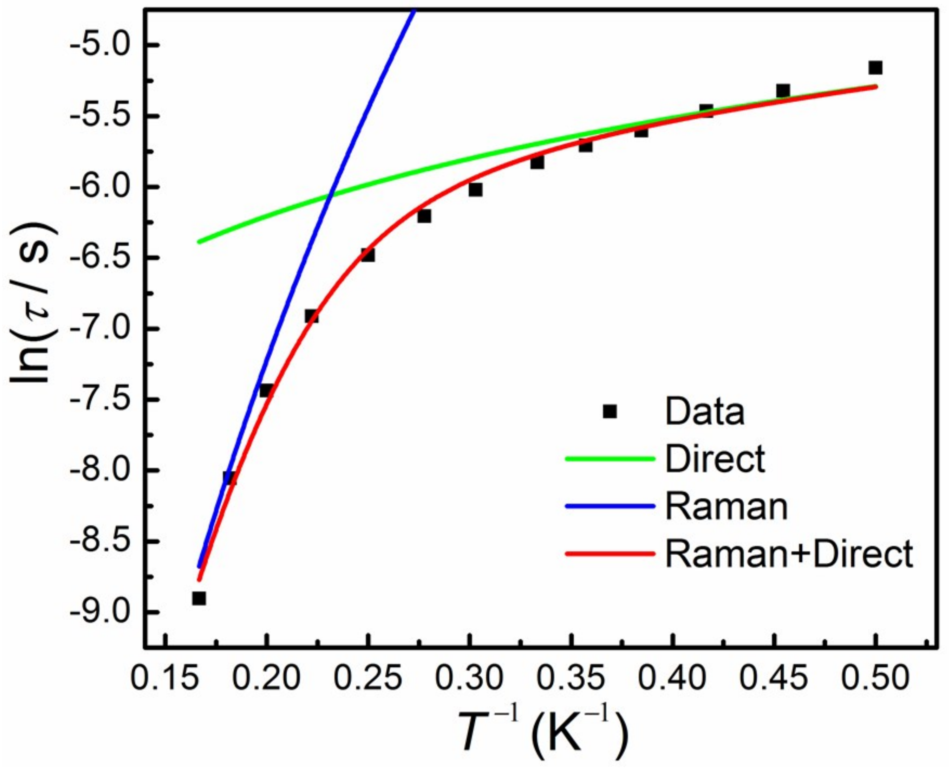

Figure S9. Temperature dependence of the magnetization relaxation rates of **1** under an applied dc field of 800 Oe. The blue and green solid lines represent the single Raman and direct mechanisms, respectively, while the red solid line represents the best fit by using a combination of the Raman and direct relaxation mechanisms.

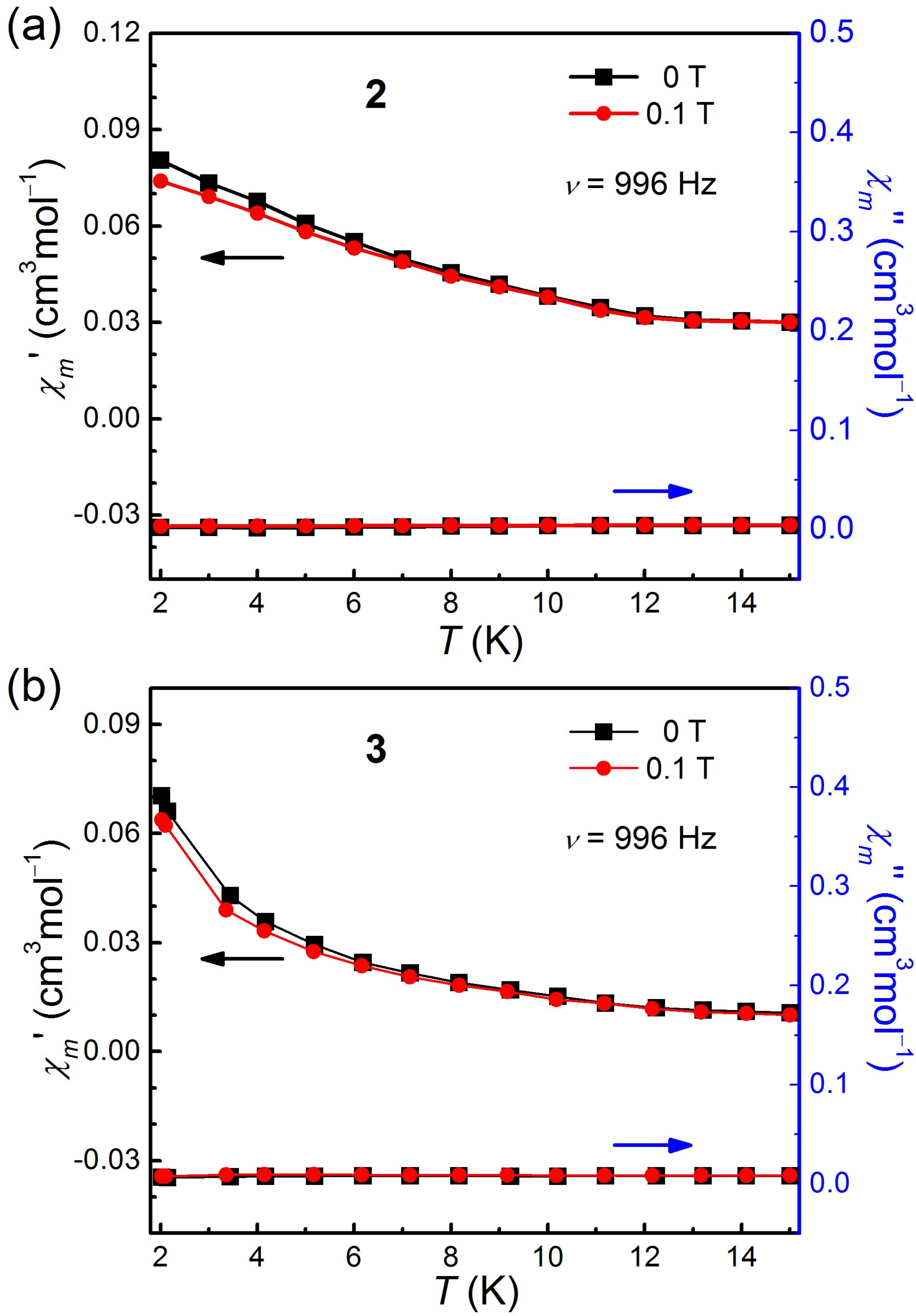

Figure S10. Temperature dependence of out-of-phase (χ'') *ac* susceptibility data for **2** (a) and **3** (b) measured under 0 and 0.1 T *dc* field.

7. References

(1) Zhang, S.-M.; Zhang, H.-Y.; Qin, Q.-P.; Fei, J.-W.; Zhang, S.-H., Syntheses, crystal structures and biological evaluation of two new Cu(II) and Co(II) complexes based on (E)-2-(((4H-1,2,4-triazol-4-yl)imino)methyl)-6-methoxyphenol. *J. Inorg. Biochem.* **2019,** *193*, 52-59.

(2) Zadrozny, J. M.; Telser, J.; Long, J. R., Slow magnetic relaxation in the tetrahedral cobalt(II) complexes $[Co(EPh)_4]^{2-}$ (EO, S, Se). *Polyhedron* **2013,** *64*, 209-217.
